# Risk in Vaccine Research and Development Quantified

**DOI:** 10.1371/journal.pone.0057755

**Published:** 2013-03-20

**Authors:** Esther S. Pronker, Tamar C. Weenen, Harry Commandeur, Eric H. J. H. M. Claassen, Albertus D. M. E. Osterhaus

**Affiliations:** 1 Vacceleron, Utrecht, The Netherlands; 2 Faculty of Economics, Erasmus University Rotterdam, Rotterdam, The Netherlands; 3 Athena Instituut, Vrije Universiteit Amsterdam, Amsterdam, The Netherlands; 4 Viroscience, Erasmus Medical Centre Rotterdam, Rotterdam, The Netherlands; University of Texas Medical Branch, United States of America

## Abstract

To date, vaccination is the most cost-effective strategy to combat infectious diseases. Recently, a productivity gap affects the pharmaceutical industry. The productivity gap describes the situation whereby the invested resources within an industry do not match the expected product turn-over. While risk profiles (combining research and development timelines and transition rates) have been published for new chemical entities (NCE), little is documented on vaccine development. The objective is to calculate risk profiles for vaccines targeting human infectious diseases. A database was actively compiled to include all vaccine projects in development from 1998 to 2009 in the pre-clinical development phase, clinical trials phase I, II and III up to Market Registration. The average vaccine, taken from the preclinical phase, requires a development timeline of 10.71 years and has a market entry probability of 6%. Stratification by disease area reveals pandemic influenza vaccine targets as lucrative. Furthermore, vaccines targeting acute infectious diseases and prophylactic vaccines have shown to have a lower risk profile when compared to vaccines targeting chronic infections and therapeutic applications. In conclusion; these statistics apply to vaccines targeting human infectious diseases. Vaccines targeting cancer, allergy and autoimmune diseases require further analysis. Additionally, this paper does not address orphan vaccines targeting unmet medical needs, whether projects are in-licensed or self-originated and firm size and experience. Therefore, it remains to be investigated how these - and other - variables influence the vaccine risk profile. Although we find huge differences between the risk profiles for vaccine and NCE; vaccines outperform NCE when it comes to development timelines.

## Introduction

Human life expectancy has increased due to the implementation of hygiene, sanitation and vaccination. Immunization strategies - of which the use of vaccines is the most important - have prevented more premature deaths, permanent disability, and suffering, in all regions in the world, than any other medical intervention [1]–[4]. Vaccines are the most cost-effective strategy with the potential to prevent - or even cure - acute and chronic infections, allergic conditions, auto-immune diseases and cancer [5], [6]. Prophylactic and therapeutic vaccination leading to both individual and herd immunity as well as symptom relief during disease progression respectively, will continue to be of fundamental value in maintaining public health in the future [7], [8].

Unfortunately, as with the pharmaceutical industry, also the biotech sector is affected by the so-called productivity gap [9], [10]. The productivity gap describes a situation within an industry whereby the invested resources do not match the expected product turn-over [9], [10]. Developing a human vaccine from the preclinical phase to registration requires an increasing average investment of approximately US$ 200 to 900 million [5]. This process is also known as the value chain; the consecutive development stages a vaccine or medical compound progresses through to accumulate value and become established as a safe, effective and qualitative product. However, merely 22% of the initiatives were forecasted in 1996 to successfully reach the market after 10 years of development [11], [12]. This imbalance is to a large extent caused by rising cost of research and development (R&D), biological and technical challenges associated with targeting more complex diseases, competition with better standards of care, larger scale of clinical studies to prove safety and efficacy and last but not least an increasingly stringent regulatory environment [13], [14]. From the perspective of the patient, and in financial terms: the subsequent attrition rate is substantial and should be improved.

Value chain descriptives - including, but not limited to; phase duration and transition rates - are important parameters for investors seeking strategic financial advice. The result of combining these two dimensions is a relatively accurate physical indication of the productivity at different development stages. The current benchmark on methodologies for determining risk profiles is published by Dimasi *et al* (2003) [15], and applies to new chemical entities (NCE). A risk profile combines data on the average phase duration with probabilities of projects transitioning between value chain phases. To date, there is limited documentation on vaccine development. Two articles in particular - one from 1996 [12], and a more recent publication from 2011 [16] - focus on value chain descriptives comparing NCE and vaccine profiles. We intend to take the analysis one step further and introduce the additional variable of the target infectious agent.

## Objectives

The present paper offers an empirical analysis on the value chain risk profiles for human vaccines in development from 1998 to 2009. We hypothesize that current vaccine risk profiles behave in a pattern similar to those described for NCEs: since 1983 to 1994 [12] the overall phase duration is postulated to have lengthened but the market entry probability is expected to remain relatively constant [17]. Moreover, by stratifying data according to infectious disease areas, we aim to identify the development stages during which attrition rates are highest. In the competitive landscape where resources are limited, the overview allows for vaccine developers and investors to anticipate common project-level challenges for the particular disease area.

## Study Design

Our methodology and assumptions are based on Struck [12], and Dimasi [17]. Data is collected on five value chain phases on the basis of availability, observing human vaccines in development from 1998 to 2009. The five development phases included in the analysis; Preclinical (PC), Human Clinical Trials Phase I–III (PI-III) and Regulatory submission (RS). Discontinued projects (D) are also included. Where available, data is updated to 2010. An active research strategy is chosen to develop the proprietary dataset, cross-referencing various sources including; commercial database (Medtrack ©), governmental sources, company sources open to the public, official press statements and scientific publications. Medtrack © is opted due to accessibility, and is compatible to Pharmaprojects ©.

Using the commercial database as a starting point, a total of 902 vaccine candidates during any stage of development were included in the dataset. Data was collected on the 12th of May 2010 showing 1495 entries. By excluding products on the market, in post-marketing trials, or where no details were found (NA), 902 unique products remain. It was assumed that the database is current and contains an accurate record of all vaccine projects, making randomization unnecessary. Consequently the data was filtered according to specific in- and exclusion criteria (Table 1). By defining the dataset we assumed that phases do not overlap, and that each vaccine progresses through the same stages in chronological order.

**Table 1:** In- and exclusion criteria for fine-tuning the dataset, inspired by [16].

Inclusion criteria	Exclusion criteria
EMPHRA Code J7 = human vaccine product, prophylactic and therapeutic.	Vaccine product undergoing post-market clinical trials for additional indications
Vaccines target human infectious diseases caused by; viral, bacteria, fungi, parasites, bacterial toxins and unspecified infectious agents.	Vaccines targeting cancer, allergy and auto-immune indications.
The database entry has descriptive information on the product; sponsor company, therapeutic area, at least one date indicating the state of the current development phase	The vaccine product cannot be found on at least one other source.Except for products in PC or D phases, as these are underreported
Product is in the following phases according to the database; PC^a^, PI^b^, PII^c^, PIII^d^, RS^e^, D^f^.	Products in the following phases according to the database: M^g^, PM^h^, NA^i^, F^j^.
Start of PC phase in 1998	Duplicate entries

a Preclinical Phase;

b Human clinical trials Phase One;

c Human clinical trials Phase Two;

d Human clinical trials Phase Three;

e Regulatory submission to allow market entry;

f Projects discontinued for any reason during any stage of the following stages of vaccine development in PC, PI, PII and PIII;

g Market phase;

h Post-marketing, also known as human clinical trials phase four;

i No information available;

j Failed or terminated vaccine products. In other words, products that have received regulatory market approval, but have been withdrawn from the market for any reason.

Phase duration was determined by the average number of years a vaccine candidate takes to complete a development phase. Discontinued projects were not included in this calculation since the decision can occur at any moment distorting results; 456 vaccine projects remain eligible. For practical reasons we consider a year to have 360 days.

The second element of the risk profile constitutes the transition probability, which was determined by applying the formula as described in [17]. Furthermore, the cumulative transition ratio is taken to represent the market entry probability. It indicates the proportion of vaccine candidates that developed successfully from PC to the highest attainable development phase. All vaccine projects in the dataset were included in these calculations.

Lastly, data was stratified according to therapeutic area for investigating this third variable influence on the phase duration and transition probabilities. Furthermore, within this infectious disease category, data on vaccines against acute infections, chronic infections, preventative indications and therapeutic indications are analyzed separately. Nevertheless, in order to recognize the significance of the disease area, risk profiles are placed into context of the disease burden and invested resources. The estimated patient population is taken to represent the former aspect, whereas the latter was measured by total sum of the value of merger and acquisition (M&A) deals. The commercial database Pharma ETrack © is consulted on financial statistics of M&A. The sum of M&A activity in US $ Million since 2004 is calculated as an indication for the amount of resources the biotech industry invests in the particular disease area. The disease areas covered by the majority of vaccine projects are presented in this paper.

## Results

**Figure 1:**
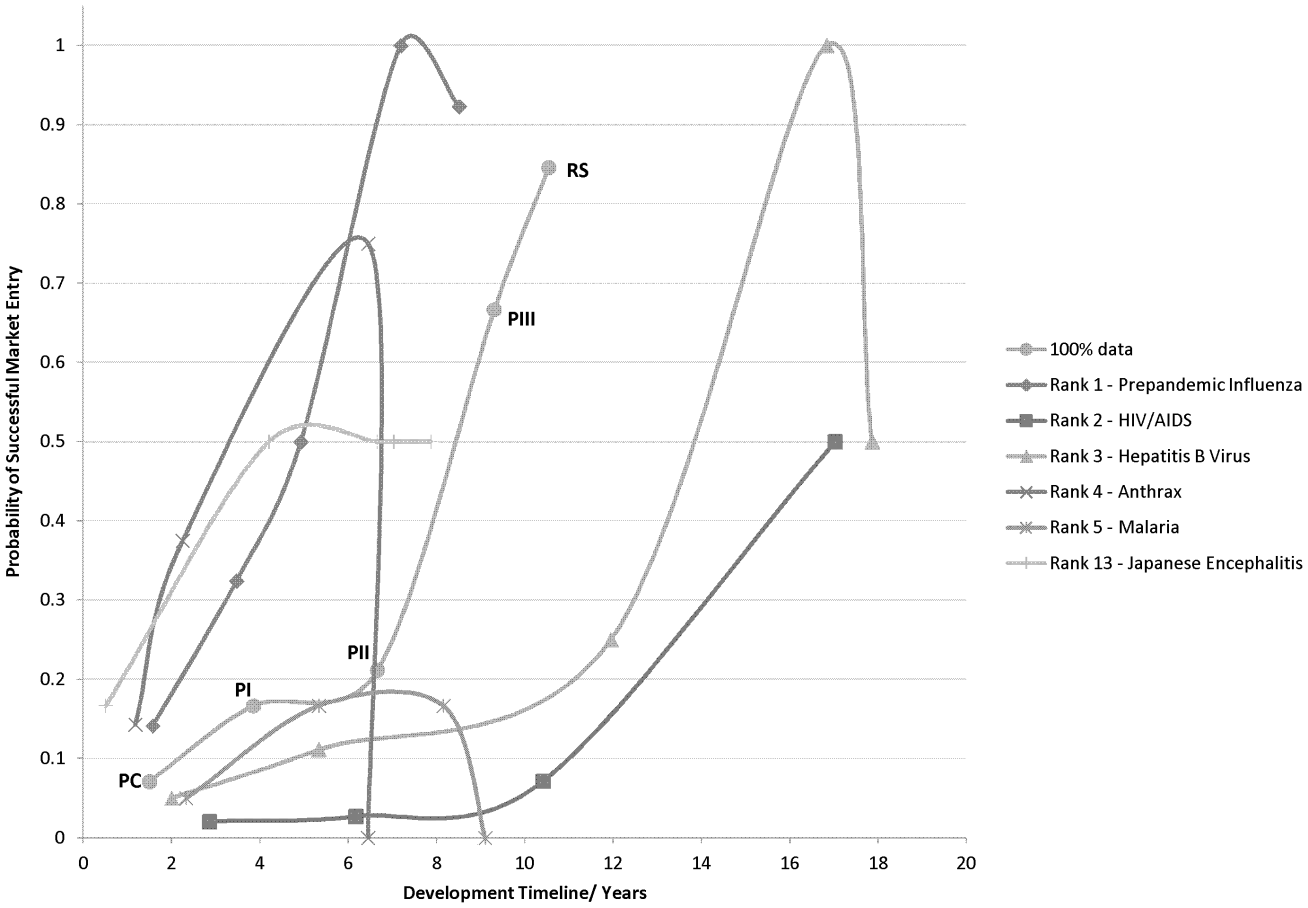
Vaccine risk profiles for selected disease areas. Risk profiles for the selected disease areas, combining phase duration with the cumulative transition probabilities as indicated by market entry probabilities. Rank order indicates quantity of projects in data set. Data points are labelled on the All Data curve; this labelling also applies to the other curves.

The filtered dataset contains 605 unique human vaccine candidates during any stage of development, from 188 individual firms covering over 60 therapeutic areas. The risk profile for the average vaccine in development from 1998 to 2009 only partially behaved as predicted (Figure 1) the timeline has lengthened by 0,71 years, yet the cumulative success rate is lower at an estimated transition probability of 0,07 (Table 2). On account of the sizeable standard deviations, we would advocate that risk profile parameters delineating the entirety of the dataset should only be interpreted as an indication for the general development trend of the vaccine manufacturing industry.

**Table 2:** Table summarizing calculations of phase duration and transition probabilities for selected vaccine target disease areas.

Disease Area	All Data					Pandemic Influenza		HIV/AIDS +^a^		HBV		Anthrax ++^b^		Malaria +		JEV	
Phase	*Duration/Yr*	*Standard Deviation* c	*High* d	*Low* d	*Transition (P)*	*Duration/Yr*	*Transition (P)*	*Duration/Yr*	*Transition (P)*	*Duration/Yr*	*Transition (P)*	*Duration/Yr*	*Transition (P)*	*Duration/Yr*	*Transition (P)*	*Duration/Yr*	*Transition (P)*
**PC**	1,50	1,89	1.73	1.27	0,07	1,57	0,14	2,86	0,02	2,00	0,05	1,18	0,14	2,32	0,05	0,51	0,17
**PI**	2,48	2,12	2.94	2.02	0,17	1,90	0,32	3,31	0,03	3,33	0,11	1,08	0,38	3,01	1,17	3,69	0,5
**PII**	2,86	2,45	3.39	2.32	0,21	1,50	0,50	4,24	0,07	6,61	0,25	4,20	0,75	2,82	1,17	2,45	0,5
**PIII**	2,64	2,19	3.45	1.84	0,67	2,26	1,00	6,61	0,50	4,89	1,00	-	-	0,95	-	0,37	0,5
**RS**	1,24	0,79	1,58	0,89	0,85	1,32	0,92	-	-	1,03	0,50	-	-	-	-	0,85	1
**% of data**	100					21,65		12,89		4,96		4,46		4,13		1,82	
**Rank based on % data**	N.A.					1		2		3		4		5		13	
**Total Duration PC-RS (Yr)**	10,71					8,51		17,02 +		17,86		6,44 ++		8,07 +		7,87	
**Disease Burden**	N.A.					1–5 Billion [35]		3,3 Million [36]		2 Billion [37]		Uncommon [28]		3–500 Million [38]		50,000 [39]	
**M&A** e	N.A.					116.000		91.120		15.600		30.700		350		77	

a + Projects do not transition from clinical trial phase III to regulatory submission phase;

b ++ Projects do not transition from clinical trials phase II to clinical trial Phase III;

c Standard deviation/year;

d 95% Confidence Interval for the mean duration/year;

e Merger and acquisition, deals in US$ Million since 2004.

When dividing the data according to the third variable - areas of therapeutic intervention - 49% of the vaccine pipeline covers 5 conditions. Furthermore, Japanese Encephalitis (JE) vaccine projects are highlighted due to multiple recent product approvals. These include; Ixiaro © (Intercell AG, also known as Jeev © and Jespect ©, available since 2009 in various countries), and beyond the scope of the dataset Encevac © (Kaketsuken, in Japan since Jan 2011) and Imojev © (Sanofi, available in Australia and Thailand since July 2011). The remaining disease areas are not represented by a sufficient quantity of vaccine projects to attain statistically significant comparisons.

By far the most lucrative business opportunity is created by pandemic influenza. In this saturated environment, efficiency is the key word if vaccine manufacturers desire to maintain a competitive advantage. As the current manufacturing capacity for influenza vaccines is limited at 900 million dosages [18], innovations are namely pursued in areas including adjuvant development, delivery system and manufacturing technology [19]. Nevertheless, pandemic influenza vaccine preparedness is a unique and rare situation that should not be compared to vaccines targeting other disease areas.

Pandemic influenza preparedness efforts are largely aligned with World Health Organization's advice to national governments on societal, antiviral and vaccine strategies, based on monitoring the threat-level of emerging potential pandemic influenza viruses [20]. Since purchasing vaccines for immunization campaigns is coordinated by national governments, they are responsible for ensuring sufficient access from the early stages of an influenza pandemic onward. Over the past few decades, several governmental bodies followed the WHO preparedness advice and proactively sought advanced purchase agreements with vaccine manufacturers. Such agreements led vaccine manufacturers to anticipate an increasing demand for influenza vaccines, and as a result they invested in expanding their manufacturing capacity. Furthermore, during the 2009 H5N1 influenza pandemic, vaccine acquisition by individual governments proved to be an inefficient system, and the European Committee (EC) responded by establishing a joint procurement initiative for future pandemic threats in November 2011 [21], [22]. Obviously the sustainability of such provisions is largely dependent on the political will and compliance of individual member states and it probably will be hard to implement in the current era of ‘post-pandemic fatigue.’

Additionally, JE represents an attractive target for vaccine developers; with merely 11 firms in our dataset investing in R&D, over 1 in 10 initiatives successfully attain regulatory submission phase. The short and steep risk profile presumes half the candidates from PC progress to subsequent PI trials. Nevertheless, due to the low number of candidate vaccines in later stages of development, we believe the timeline is underestimated.

The foremost challenge in vaccine development is reducing the average transition rate from clinical phase II to III. Between these value chain phases the risk profile incorporating all data has an estimated transition probability of 0,21. It represents the highest attrition rate when compared to the productivity of the other phases. Both Anthrax and Malaria risk profiles confirm the bottleneck, as project development activities do not advance beyond PIII. The phenomenon has been recognized in NCE development, and we believe the underlying mechanisms and explanations are also applicable for vaccines [23]–[25].

A second major obstacle is a successful transition from clinical phase III to regulatory submission. Such bottlenecks are evident in Pandemic Influenza, and Hepatitis B vaccine (HBV) risk profiles, whereby the attrition rate for HBV candidates is calculated at an astonishing 50%. HIV/AIDS projects are also affected by significant attrition rates between these stages. Reasons for submission failures have been described for NCEs, and we assume similar arguments are relevant [26].

**Figure 2:**
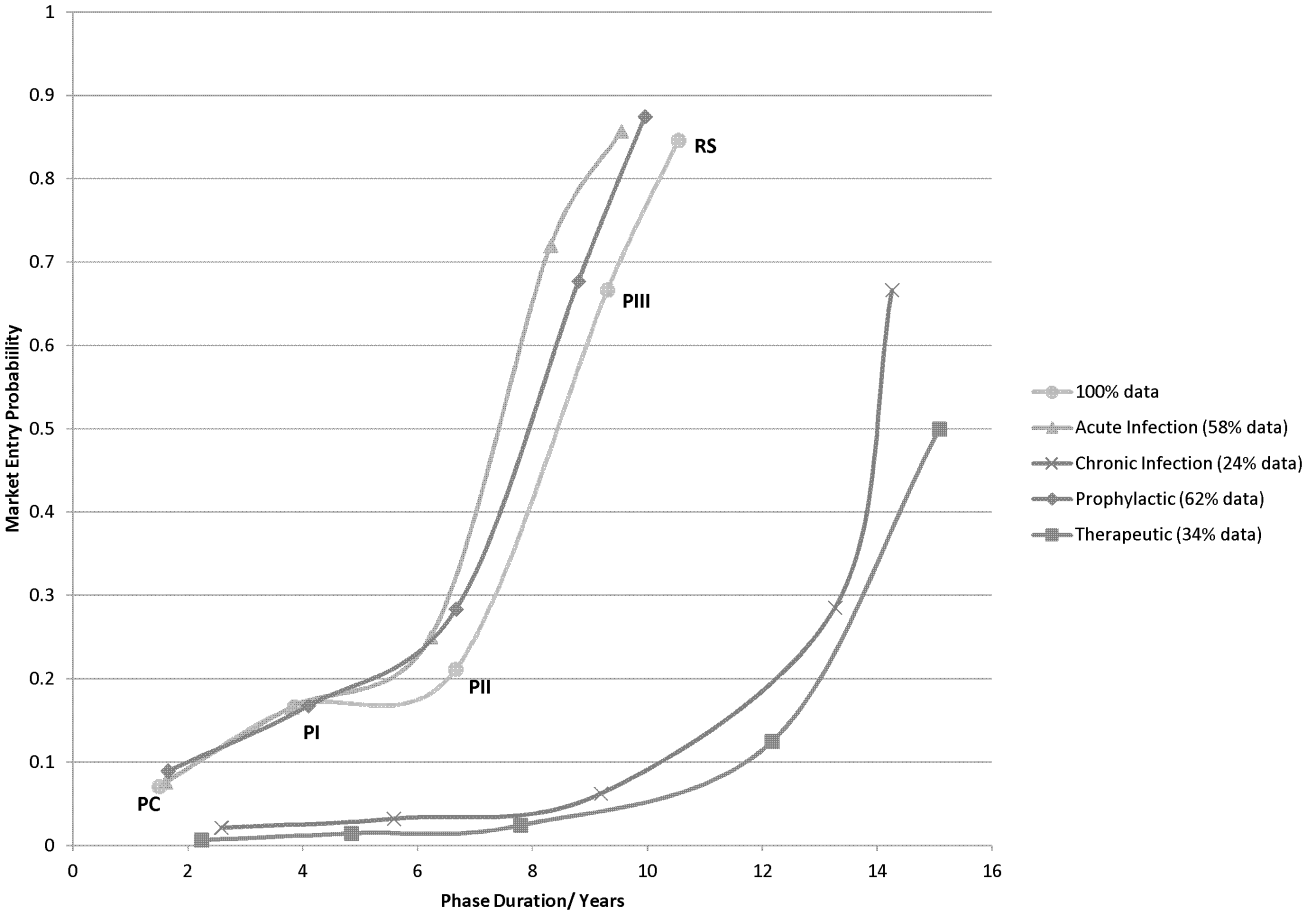
Vaccine risk profile granulating acute versus chronic infections and prophylactic versus therapeutic vaccines. Risk profiles for vaccines, granulated to show risk profiles for vaccines targeting acute versus chronic infections and prophylactic versus therapeutic vaccines. Groups are stratified from 100% of the data from the dataset. Percentage per group included.

Data was further granulated into vaccines targeting acute versus chronic indications and prophylactic versus therapeutic applications (Fig 2). According to the dataset, vaccines targeting acute infectious diseases, as well as prophylactic vaccines clearly have a lower risk profile when compared to vaccines targeting chronic diseases or therapeutic applications.

**Figure 3:**
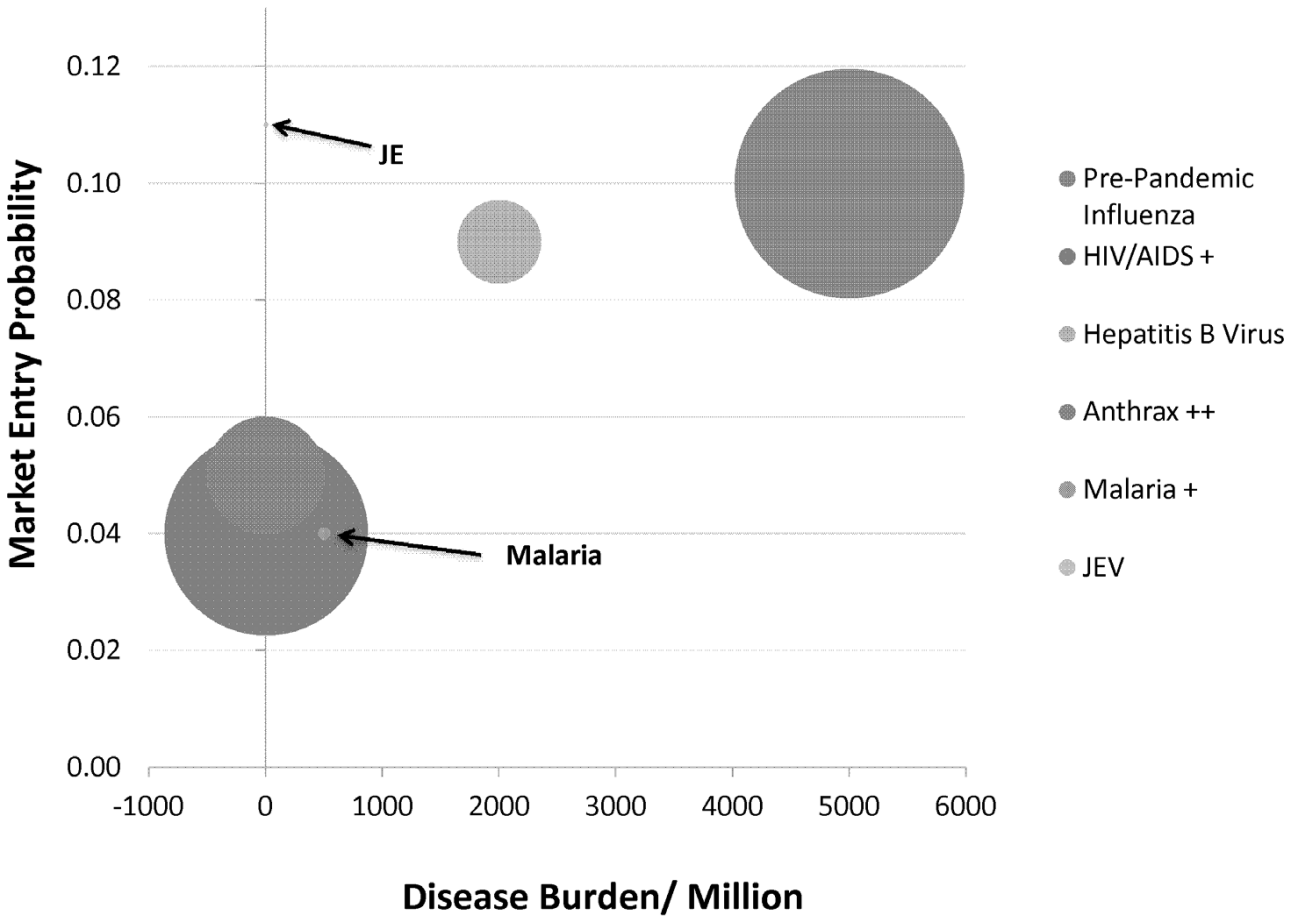
Combining the cumulative success rate with contextual factors. Combining the cumulative success rate with the contextual factors of disease burden and size of investment (indicated by the size of the bubble).

As a final observation: it is generally assumed that a higher disease burden - preferably in the industrialized world [27] - is an incentive to dedicate resources to that particular disease area (Fig 3; Table 2). As an example of the opposite being the case sometimes: 4% of vaccine development projects target anthrax, while the infection is highly uncommon and related to biological-warfare [28].

## Conclusion

Risk profiles are important descriptive tools providing indications on possible future vaccine project outcomes, essential for strategic decision making. In general, the more recent vaccine development projects from 1998 to 2009 showed a longer timeline with a lower probability of market entry than those from 1983 to 1994 [12]. What could partially explain the increased phase length could be the fact that the ICH-E6 Good Clinical Practice guidelines came into effect after Struck's publication in 1996, which has influenced clinical research on a global scale. However, we feel that the phase lengths calculated are not fully representative for the actual situation. Certain preliminary R&D activities - such as *in silico* lead selection and toxicity screening - taking place prior to patenting are not represented in the dataset. We believe that when these procedures are taken into consideration, the actual development timeline is expected to be even longer still.

Additionally, the lengthening timelines for vaccine development may be influenced by the fact that the so-called ‘low-hanging fruits’ has already been picked. Data confirms that the majority of vaccine R&D projects encompassing incremental innovations targeting disease areas with known correlates of protection have a shorter development timeline when compared to more radical vaccine innovations [29]. Nevertheless, vaccine timelines remain significantly shorter when compared to NCE development.

Clarifications for the discrepant transition probabilities between our dataset and previous articles expediently relate to data collection methodologies. Moreover, stratifying data according to acute or chronic indications as well as therapeutic or prophylactic application revealed significant variations in transition success. This confirms that one risk profile cannot represent the productivity of the overall vaccine development field, and the effect of a third confounding variable is essential information.

Essentially, infectious diseases are different from cancer, and both are fundamentally different from allergy and autoimmune diseases with respect to the mechanisms of pathogenesis, immunity as well as the approach and difficulty of vaccine development. Consequently, vaccine development for infectious diseases, cancer, and allergy/autoimmunity should be analyzed separately. Moreover, this paper does not address orphan vaccines targeting unmet medical needs, whether projects are in-licensed or self-originated and firm size and experience [14], [30], [31]. Therefore it remains to be investigated how these other variables influence the vaccine risk profile.

Vaccine development is a risk intensive exercise and requires substantial investments. As indicated by the risk profiles: the ratio of success to failure is in favour of the latter. Both the burden of disease and the magnitude of invested resources into a project targeting a specific infectious agent do not correlate with a higher success rate. Substantially resources are dedicated to HIV/AIDS, even though within the scope of our dataset there are no regulatory approved vaccines. It is interesting to note that preventive vaccine development against JEV - a virus that causes acute infection - may not be considered such a lucrative target as the market size is too limited to guarantee a rapid return on investment. Obviously other criteria are used for vaccine target selection [32], [33]. These high rates of attrition need to be reduced in order to sustain business case growth [34] and respond appropriately to public health demands.

Several considerations apply to this study. First, we have assumed that the database on commercial vaccine development - on which the dataset is based - keeps an accurate record of all vaccine development projects currently in any phase of development. The dataset we compiled is unique; however the explicit delineation of methodologies should allow other research groups to replicate procedures. Additionally, phase lengths are influenced by the spread of the data points. The majority of the vaccine development dates (>50%) were collected after the year 2000 implying the spread of points is not equally distributed within the dataset. This either suggests that the earlier years of the selected timeframe are underrepresented, or that the actual quantity of projects has increased over the years.

This paper provides a descriptive historical account of vaccine development between 1998 and 2009, and does not have the ambition to forecast any trends. Moreover we have not addressed the numerous reasons that may lead to project termination, and do not disregard the necessary legislative requirements in the development of ethical, safe, effective, and high quality vaccines.
